# Accurate extraction of surface water in complex environment based on Google Earth Engine and Sentinel-2

**DOI:** 10.1371/journal.pone.0253209

**Published:** 2021-06-18

**Authors:** Jianfeng Li, Biao Peng, Yulu Wei, Huping Ye

**Affiliations:** 1 Institute of Land Engineering and Technology, Shaanxi Provincial Land Engineering Construction Group Co., Ltd., Xi’an, China; 2 Shaanxi Provincial Land Engineering Construction Group Co., Ltd., Xi’an, China; 3 Key Laboratory of Degraded and Unused Land Consolidation Engineering, The Ministry of Natural Resources, Ltd., Xi’an, China; 4 Shaanxi Provincial Land Consolidation Engineering Technology Research Center, Ltd., Xi’an, China; 5 State Key Laboratory of Resources and Environmental Information System, Institute of Geographic Sciences and Natural Resources Research, Chinese Academy of Sciences, Beijing, China; Duy Tan University, VIET NAM

## Abstract

To realize the accurate extraction of surface water in complex environment, this study takes Sri Lanka as the study area owing to the complex geography and various types of water bodies. Based on Google Earth engine and Sentinel-2 images, an automatic water extraction model in complex environment(AWECE) was developed. The accuracy of water extraction by AWECE, NDWI, MNDWI and the revised version of multi-spectral water index (MuWI-R) models was evaluated from visual interpretation and quantitative analysis. The results show that the AWECE model could significantly improve the accuracy of water extraction in complex environment, with an overall accuracy of 97.16%, and an extremely low omission error (0.74%) and commission error (2.35%). The AEWCE model could effectively avoid the influence of cloud shadow, mountain shadow and paddy soil on water extraction accuracy. The model can be widely applied in cloudy, mountainous and other areas with complex environments, which has important practical significance for water resources investigation, monitoring and protection.

## Introduction

With the rapid development of aerospace technology, remote sensing technology provides advanced methods for many fields such as resource survey, environmental monitoring, regional analysis, and global macro research [1–3]. Accurate extraction of water information from remote sensing images is of great significance to watershed planning and investigation, monitoring and protection of water resources [4, 5].

Sri Lanka has emerged as a maritime hub connecting Asia-Africa-Europe shipping routes; this is of strategic, economic, and geopolitical significance to the country. The water environment of the island is complex and includes many types of water bodies, such as lakes, reservoirs, pit-ponds, lagoons and rivers. Sri Lanka is a tropical region, resulting in earth observation satellite images are affected by cloud cover and cloud shadow all the year round, and cloud-free images account for less than 1%. At the same time, there are many paddy fields growing rice in the island, and the spectral characteristic of water is close to those of the soil after harvesting rice, especially after rain. These factors increase the difficulty of obtaining accurate surface water information from remote sensing image.

In recent years, scholars around the world have carried out extensive research on the extraction of water from images of different satellite sensors. According to the relationship between the second and fourth bands of TM image, Mcfeeters et al. proposed the normalized difference water index (NDWI), which can effectively suppress soil and vegetation information, but it is difficult to suppress building information [6]. Zhou et al. found that water body in TM image has unique characteristic of spectral relationship, namely (TM2+TM3) > (TM4+TM5), which is particularly suitable for the extraction of water in mountainous areas [7]. On the basis of NDWI, Xu introduced the modified normalized difference water index (MNDWI), which shows higher accuracy, especially in urban water extraction [8]. Yan et al. proposed the enhanced water index (EWI), and combined with GIS technology to effectively extract surface water in semi-arid areas [9]. Zhang et al. constructed an automatic water extraction model based on the characteristic that the greenness component and the fourth component of the water in TM images are less than the wetness component after K-T transformation [10]. Based on Landsat-8 OLI image, Zhang et al. constructed a water extraction model based on LBV transformation, which has better accuracy than NDWI and MNDWI [11]. Jiang et al. applied the multilayer perceptron neural network to the extraction of surface water from Landsat-8 OLI image, which effectively suppressed the influence of shadow, ice and snow [12]. Combining Sentinel-2 image and OpenStreetMap data, Zhang et al. proposed a water body extraction model based on presence and background learning algorithms, and proved the model has higher accuracy than the exponential of water body and random forest through experiments [13]. Scholars around the world have proposed many water extraction models for different sensors, mainly focusing on conventional environment, large-scale and high-efficiency water extraction [1, 14, 15], and rarely pay attention to the accurate extraction of water in complex environment. However, in cloudy, mountainous, and paddy soil areas such as those Sri Lanka, the conventional models cannot effectively eliminate the influence of cloud shadows, mountain shadows, and paddy soil on the accuracy of water extraction, so the accurate extraction of surface water is faced with challenges.

The Sentinel-2 satellite is the second satellite in the Copernicus programme of the European Commission (EC) and the European Space Agency (ESA) [16]. The main task of Sentinel-2 satellite is to realize global land surface resolution multispectral imaging. Compared with Landsat-8 image, Sentinel-2 image has higher spatial and temporal resolution. In terms of water extraction using water body index (NDWI, MNDWI), the accuracy of Sentinel-2 image is better than Landsat-8 image, especially in river extraction [17]. The Sentinel-2 image has blue, green, red, and near-infrared bands with a resolution of 10 m, and the resolution of the two short-wave infrared bands commonly used as input bands of water index is 20 m, which reduces the accuracy of the water extraction results to some extent. NDWI only uses the green and near-infrared bands with a resolution of 10m, which has been proved to have obvious misclassification error [18]. The band sharpening method can extract water with an accuracy of 10m [19], but it requires a lot of time-consuming calculations. Wang et al. selected four 10m bands and two 20m shortwave infrared bands of Sentinel-2 image as input features, and trained through SVM to find the optimal separation hyperplane, and proposed the Multi-Spectral Water Index (MuWI), which achieved surface water resources mapping with 10m resolution [20]. However, MuWI is difficult to be universal due to the limitation of training sample size. To achieve high-precision water extraction in complex environment, an AWECE water extraction model is constructed based on Google Earth Engine remote sensing cloud computing platform and Sentinel-2 image. On the basis of analyzing the differences of spectral characteristics of typical ground objects, three sites were selected from Sri Lanka to test the water extraction accuracy of AWECE, NDWI, MNDWI and MuWI-R models.

## Materials and methods

### Study area

**Fig 1:**
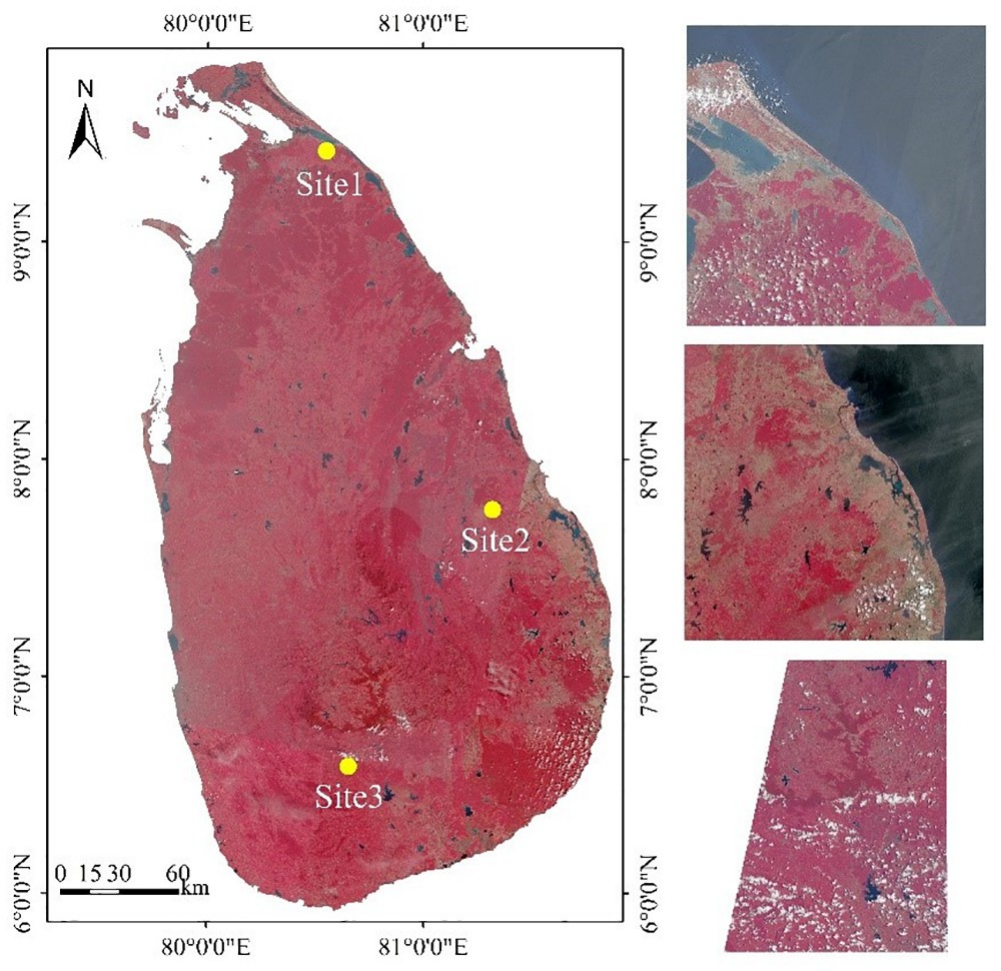
Sentinel-2 false-color composite image of study area (8, 4, 3 bands).

Sri Lanka, an island of Indian ocean, is located in the south of the South Asian Sub-Continent and southeast of India, with a tropical monsoon climate. The island measures about 224 km from east to west, and about 432 km from south to north, with a total land area of about 65610 km^2^. As the central mountains block the warm and humid southwest monsoon, only the southwest of the island is humid due to frontal rain, and most of the areas are arid or semi-arid. During the monsoon season, there is a short dry season in January and February in the humid region, with plenty of rain in the rest of the season. In arid areas, there are obvious wet (October to February) and dry seasons. According to the distribution characteristics of surface water in Sri Lanka, three sites were selected to test the accuracy of the water extraction model, including various types of water. Site 1 is located in the northern plains of Sri Lanka; the eastern coastal area of site 2 has a large number of rice-growing areas, and the southwest is a high mountain area; site 3 is located in the south-central area, with high mountains in the north and plains in the south. The false-color composite image of Sentinel-2(8, 4, 3 bands) in the study area is shown in Fig 1.

### Data

Sentinel-2 belongs to high resolution multispectral imaging satellite, which consists mainly of two satellites including 2A and 2B. Sentinel-2A was launched on 23 June 2015 and Sentinel-2B has been in orbit since 7 March 2017, with a revisiting cycle of approximately 5 days. Sentinel-2 carries a multi-spectrometer whose 13 spectral bands span visible, near-infrared and short-wave infrared. The image is freely available at Sentinel Scientific Data Hub (https://scihub.copernicus.eu). The Sentinel-2 data of this study come from the "COPERNICUS/S2_SR" dataset provided by Google Earth Engine. "COPERNICUS/S2_SR" is obtained by running the Sen2cor plug-in provided by ESA, which belongs to the L2A level and is mainly subjected to atmospheric correction. Rice is the main food crop in Sri Lanka, with two seasons a year. March and September are the harvest seasons for rice. When the rice is harvested, the soil will become wetter due to rainfall, which will affect the extraction results of the surface water. To test the water extraction accuracy of different models at different sites, a scene of Sentinel-2 image was selected for each site. The factors that affect the accuracy of water extraction are different in different sites. Site 1 is mainly cloud shadow, site 2 is cloud shadow and paddy soil, and site 3 is cloud shadow and mountain shadow. Sentinel-2 image in September is selected for site 2, and the eastern coastal area contains a large number of paddy soils. Site 1 and site 3 randomly selected images. Specific information about selected images is shown in Table 1.

**Table 1:** Specific information of selected Sentinel-2 images.

Site	Date	Sensor	Cloud Cover	Main Water Types
Site 1	2019/07/25	Sentinel-2B	1.66%	ocean, lake, reservoir, pit-pond, lagoon
Site 2	2018/09/05	Sentinel-2B	3.39%	ocean, lake, reservoir, river, pit-pond, lagoon
Site 3	2019/04/03	Sentinel-2B	6.56%	lake, reservoir, river, pit-pond

## Methodologies

### Google Earth Engine

Google Earth Engine is a cloud platform provided by Google for online visual computing and analysis of global-scale geoscience data [21]. The platform mainly stores satellite images and other earth observation data, and provides enough computing power to transfer and process the stored data. Compared with traditional image processing software such as ENVI and Erdas, Google Earth Engine can process images quickly and in batches, and the analysis results can be obtained without downloading image data. The platform provides online JavaScript API and offline Python API, and web services based on Google cloud can be quickly built by calling API.

### Sentinel-2 cloud mask based on SVM and cloud scoring algorithm

Cloud mask processing is an important step in the pre-processing of water body extraction using remote sensing images, especially in tropical areas such as Sri Lanka. QA60 band [22] or cloud scoring algorithm [23] can be used to detect clouds in Sentinel-2 image on Google Earth Engine platform. The QA60 band is generated by the blue band and two short-wave infrared bands of the Sentinel-2 image, but the effect of cloud detection is unstable, which usually leads to underestimation or overestimation [24, 25]. The main principle of the cloud scoring algorithm is to use the combination of brightness, temperature and normalized difference snow index (NDSI) to calculate the cloud possibility score. The higher the score of a single pixel, the greater the possibility of belonging to the cloud. Complex atmospheric conditions and the lack of thermal infrared bands in Sentinel-2 images generally cause cloud scoring algorithms to underestimate the range of clouds [24, 26].

**Fig 2:**
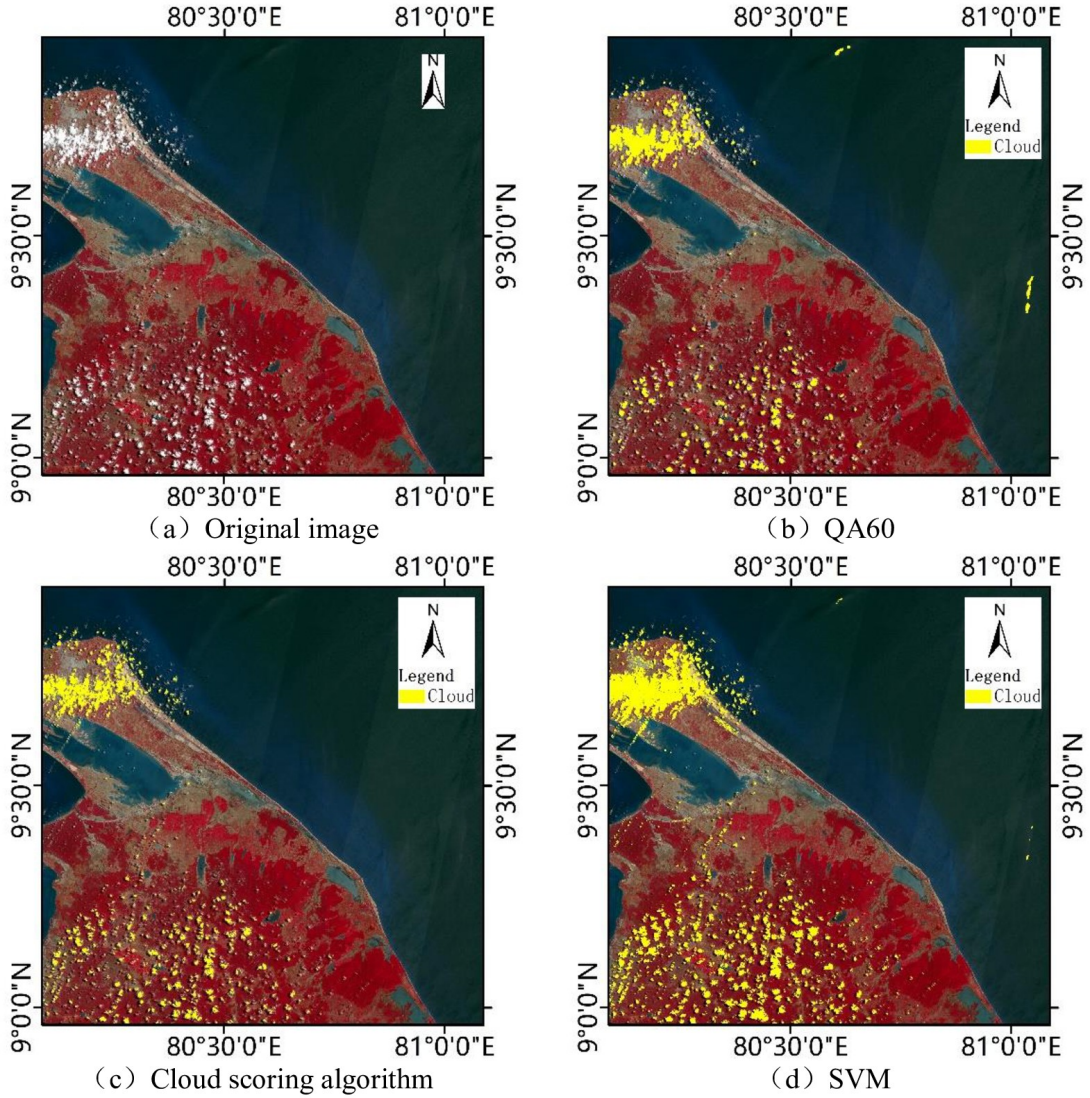
Cloud detection results of site 1 with different methods.

Compared with the threshold based cloud detection algorithm, the machine learning algorithm is better for cloud detection [27]. In order to identify clouds more accurately, the results of cloud scoring algorithm are used as training samples of SVM [28]. Samples are screened by random sampling, and finally the range of clouds is obtained by classification. Taking the selected image of site 1 as an example, QA60 band, cloud scoring algorithm and SVM are used to detect cloud respectively and the results are shown in Fig 2. It can be seen from Fig 2 that both QA60 band and cloud scoring algorithm have the phenomenon of missing extraction, especially for thin clouds. The QA60 band even experienced over-extraction, which misclassified some clear pixels at sea as clouds. Compared with QA60 band and cloud scoring algorithm, SVM has the best cloud detection results and can accurately identify the cloud boundary.

### K-T transformation of Sentinel-2 image

K-T transformation (also called Tasseled cap transformation) [29] is a special principal component analysis (PCA), which was proposed by Kauth and Thomas. K-T transformation makes the coordinate axis point to the direction closely related to the characteristics by rotating the coordinate space, especially closely related to the plant growth process and the soil. It is helpful to explain and analyze crop characters. The first component brightness of K-T transformation represents the total radiation energy level, the second component greenness reflects the growth status of vegetation, and the third component wetness reflects the humidity information of ground objects. The wetness component is the most sensitive to soil wetness information and it is a better characteristic band for water information extraction. Nedkov deduced the conversion coefficients of brightness, greenness and wetness of K-T transformation of Sentinel-2 image in 2017 [30, 31]. The K-T transformation has been used to extract water from Landsat images [31–33]. The K-T transformation equations of different sensor images can be derived by Gram-Schmidt orthogonalization (GSO) or Procrustes Analysis (PCP) method. Table 2 shows the K-T transformation coefficients of Sentinel-2 images derived by Nedkov [34] using GSO and SHI [35] using PCP method.

**Table 2:** K-T transformation coefficients of Sentinel-2 images.

Bands	GSO			PCP		
	B	G	W	B	G	W
B1-Coastal	0.0356	-0.0635	0.0649	0.2381	-0.2266	0.1825
B2-Blue	0.0822	-0.1128	0.1363	0.2569	-0.2818	0.1763
B3-Green	0.136	-0.168	0.2802	0.2934	-0.3020	0.1615
B4-Red	0.2611	-0.348	0.3072	0.3020	-0.4283	0.0486
B5-RE-1	0.2964	-0.3303	0.5288	0.3099	-0.2959	0.0170
B6-RE-2	0.3338	0.0852	0.1379	0.3740	0.1602	0.0223
B7-RE-3	0.3877	0.3302	-0.0001	0.4180	0.3127	0.0219
B8-NIR-1	0.3895	0.3165	-0.0807	0.3580	0.3138	-0.0755
B8A-NIR-2	0.4750	0.3625	-0.1389	0.3834	0.4261	-0.0910
B9-WV	0.0949	0.0467	-0.0302	0.0103	0.1454	-0.1369
B10-Cirrus	0.0009	-0.0009	0.0003	0.0020	-0.0017	0.0003
B11-SWIR-1	0.3882	-0.4587	-0.4064	0.0896	-0.1341	-0.7710
B12-SWIR-2	0.1366	-0.4064	-0.5602	0.0780	-0.2538	-0.5293

B: Brightness; G: Greenness; W: Wetness; RE: Vegetation red edge; WV: Water vapour.

**Fig 3:**
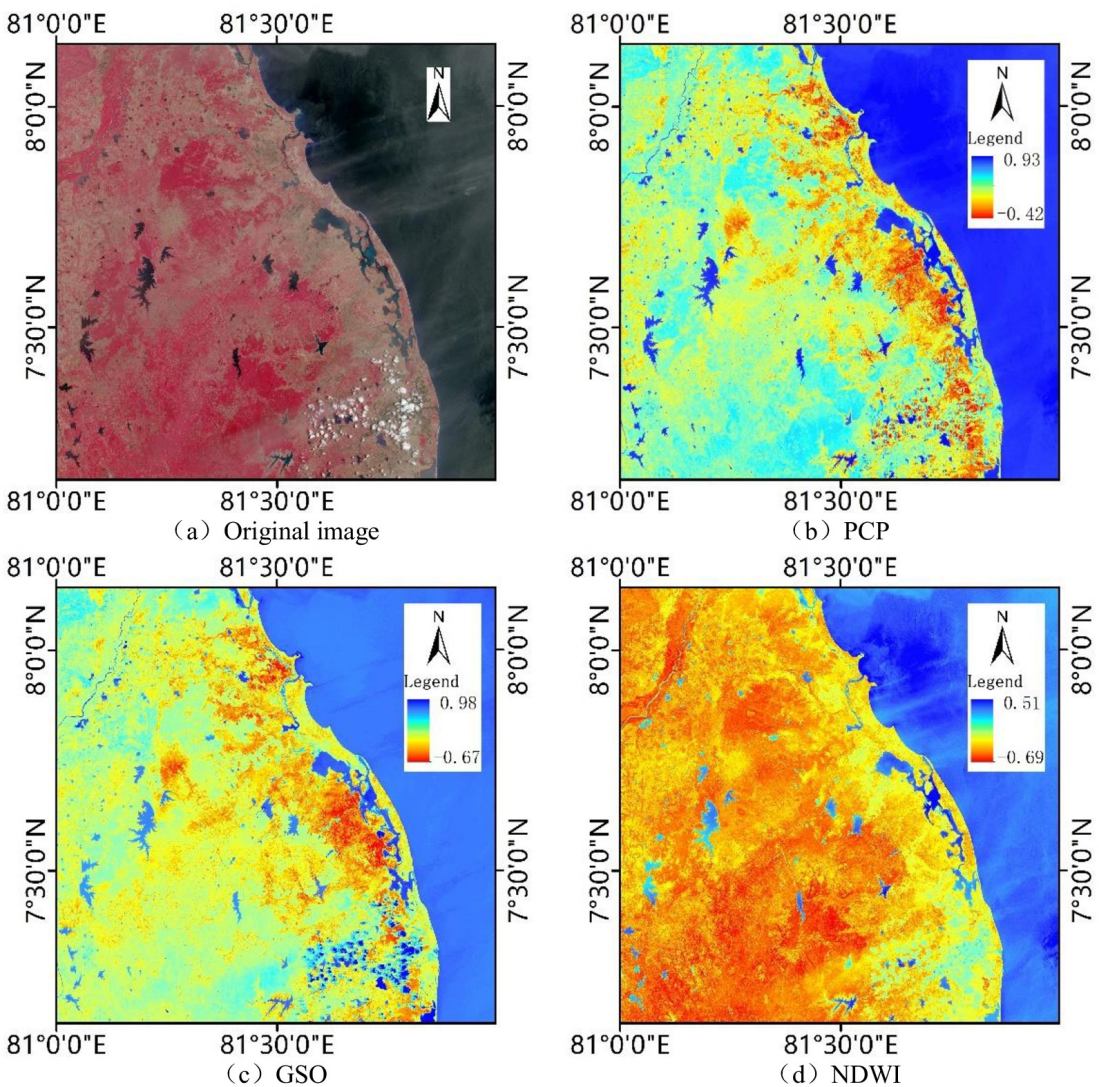
The original image, wetness component of the K-T transformation and NDWI of site2.

Taking the image selected by site 2 as an example, based on the K-T transformation coefficients (Table 2) and IDL8.5 platform, the K-T transformation result of the image is obtained, and the value is stretched. Fig 3 shows the original image, wetness component of the K-T transformation and NDWI of site2. Through comparison, it can be found that the effect of PCP method is better, and there are obvious differences between surface water and other ground objects. The wetness component values of different types of water have a small span, and all appear dark blue, with more prominent boundaries. The wetness component performance of the GSO method is poor, and the wetness component values of different types of water have a large span. The color of the water image is from light blue to dark blue, and some thick clouds are also dark blue. In addition, the boundaries of some water bodies are not significantly different from other features, especially rivers. The main reason for the poor performance of the wetness component derived by the GSO method is that the cumulative error is produced in the iterative calculation process, and the cumulative error is proportional to the number of bands of the image. The Sentinel-2 image has 13 bands, so the effect of the wetness component is poor. The color of the water body in the NDWI image is from light blue to dark blue. Some cloud shadows and paddy soils also appear light blue, and the rivers are extremely discontinuous. In contrast, the difference between surface water and other ground objects in the wetness component of K-T transformation derived by PCP method is more obvious. Therefore, the K-T transformation coefficients of sentinel-2 image derived by SHI [35] is selected in this study.

**Fig 4:**
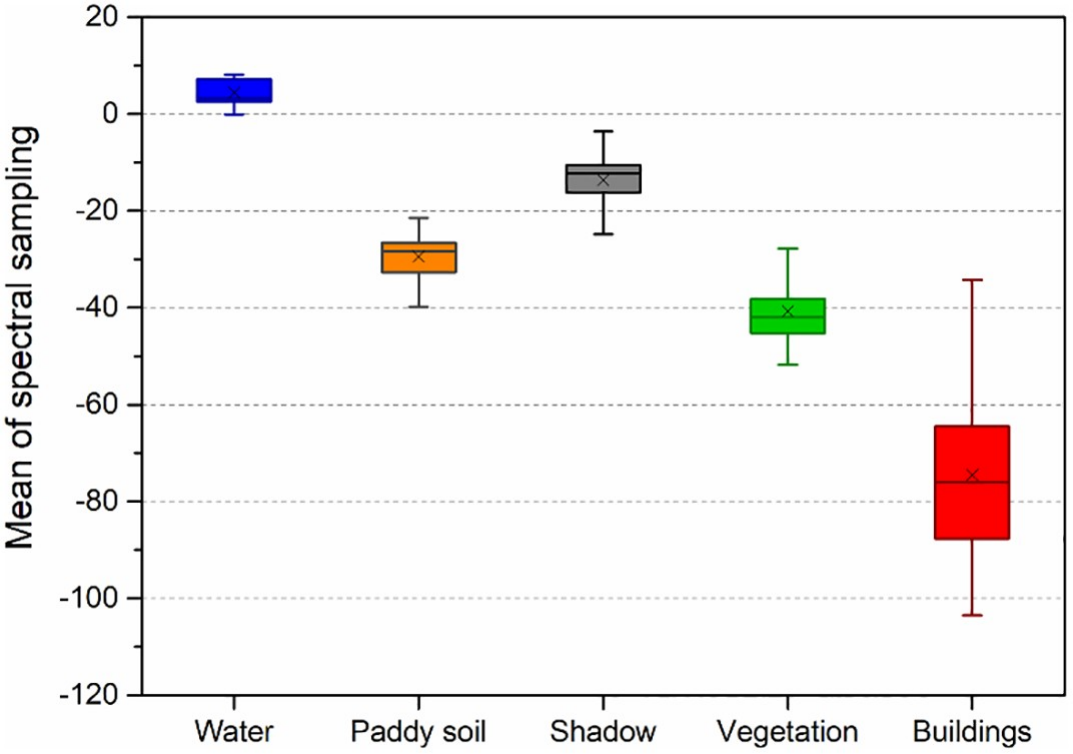
K-T transformation wetness component boxplot of typical features.

To further quantify the difference between water and other ground objects, 100 samples were selected respectively for five typical features of water, vegetation, building, shadow, and paddy soil in three sites, and the value of wetness component after K-T transformation was calculated. Fig 4 shows the boxplot of the value of the wetness components of five typical features. It can be seen from the figure that the mean value of wetness component of the water is obviously higher than that of the other 4 typical features, especially vegetation and buildings, and the span of the value is small. Even the minimum value is significantly higher than the maximum value of shadow and paddy soil. The above analysis also proves that the wetness component of K-T transformation is an outstanding characteristic band for water information recognition.

### OTSU

OTSU [36] is a widely used dynamic threshold determination algorithm, which can realize adaptive dynamic determination of image threshold by maximizing the class variance and not affected by image contrast and brightness [37]. Through the idea of clustering, OTSU divides the gray value of the image into two parts according to the gray level, so that each part has the minimum gray difference, and the difference between the two parts is the largest. By calculating the variance, we can find a more appropriate gray level to divide. The calculation process of the optimal threshold t of the OTSU algorithm is as follows:

{δ2=Pnw∙(Mnw-M)2+Pw(Mw-M)2M=Pnw∙Mnw+Pw∙MwPnw+Pw=1t=ArgMaxx≤t≤y{Pnw∙(Mnw-M)2+Pw(Mw-M)2} (1)

*δ* is the inter-class variance of non-water and water; *P*_*nw*_ and *P*_*w*_ are the possibility that a single pixel belongs to non-water and water; *M*_*nw*_ and *M*_*w*_ are the average gray level of non-water and water pixel; *M* is the average gray level of image pixel.

### Automatic water extraction model in complex environment (AWECE)

**Fig 5:**
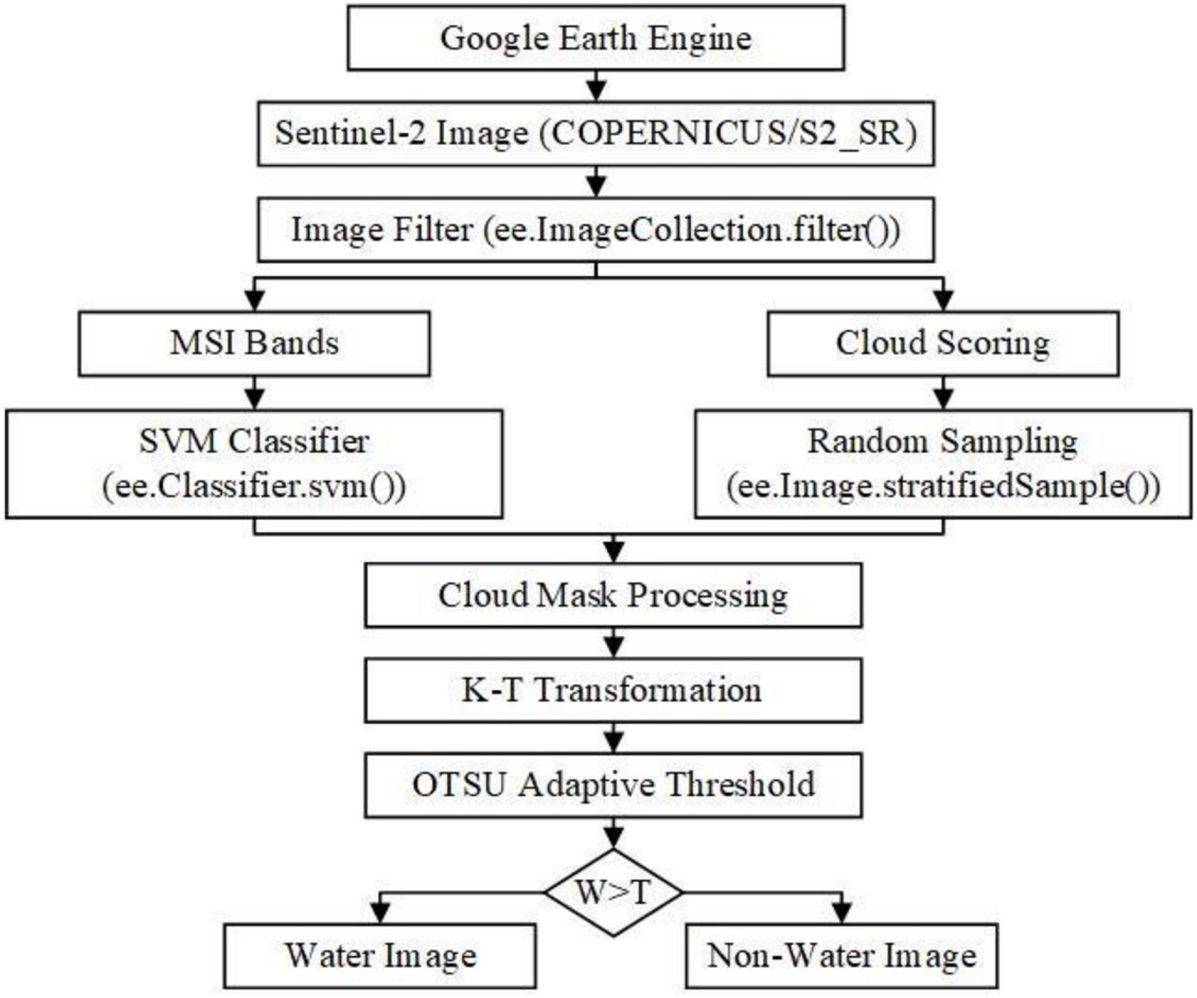
Flow chart of the AWECE.

Based on Google Earth Engine, coupled with SVM and cloud scoring algorithm for cloud detection of Sentinel-2 images, combined with the wetness component of K-T transformation, this study constructed an automatic water extraction model in complex environment (AWECE). Fig 5 shows the implementation process of AWECE. First, the time, location, and other information are taken as the conditions to determine the image of surface water to be extracted by coding on Google Earth Engine. Second, SVM (ee.Classifier.svm()) is used to train the cloud detection results of the cloud scoring algorithm after random sampling (ee.Image.stratifiedSample()). The cloud range is obtained by classification and used to mask the Sentinel-2 multispectral bands. Then, the wetness component of the image is obtained by the K-T transformation, and threshold T is adaptively determined by the OTSU algorithm. Finally, the water and non-water pixels are separated according to the threshold T. If the pixel value in the wetness component is greater than T, then it is water, otherwise it is non-water.

### Spectral water index method

The principle of spectral water index method is mainly based on the differences of spectral characteristics of water bodies in different bands, which is constructed by calculating the ratio of high reflectivity band to high absorptivity band, and then the water information is extracted by threshold segmentation [38]. The spectral water index method enhances the image features, reduces the influence of the environmental conditions around the water, and increases the difference between the water and other features. In this study, NDWI [6], MNDWI [8] and MuWI-R [20] are selected to study, and the expression is shown in Table 3.

**Table 3:** Expression of NDWI, MNDWI and MuWI-R.

Water Index	Equation
NDWI	$NDWI=\frac{\rho_{Green}-\rho_{NIR}}{\rho_{Green}+\rho_{NIR}}$
MNDWI	$MNDWI=\frac{\rho_{Green}-\rho_{SWIR}}{\rho_{Green}+\rho_{SWIR}}$
MuWI-R (Sentinel-2)	MuWI-R = −4*ND*(2, 3) + 2*ND*(3, 8) + 2*ND*(3, 12) − *ND*(3, 11)

### Accuracy assessment

Confusion matrix [39], also known as the error matrix, is a comparison array used to represent the number of pixels divided into a certain category and the ground test as this kind of number. It has been widely used in the classification accuracy evaluation of remote sensing images. Each column of the confusion matrix represents the prediction category, and the total number of each column represents the number predicted for that category; each row represents the true attribution category of the data, and the total number of data in each row represents the real number of the category. According to the confusion matrix, a variety of indicators to evaluate the classification accuracy of the model can be obtained, such as overall accuracy, commission error, omission error and so on. The confusion matrix is defined as:

$$A=\left[\begin{array}{ccc} a_{11} & \cdots & a_{1n}\\ \vdots & \ddots & \vdots \\ a_{n1} & \cdots & a_{nn} \end{array}\right]$$ (2)

The overall accuracy represents the proportion that the whole test sample is correctly classified by the model, that is, the number of samples correctly classified by the model is divided by the total number of test samples.


$$Accuracy=\frac{\sum_{i=1}^{n}a_{ii}}{\sum_{i=1}^{n}\sum_{j=1}^{n}a_{ij}}$$ (3)

The commission error indicates the proportion of the sample which is divided into a certain kind of surface features but does not belong to this kind of surface features in all samples. The misclassification error is the proportion of the sample which is divided into a certain kind of surface features but not actually belongs to this kind of surface features in all samples.

## Results

### Spectral characteristic analysis of typical features

**Fig 6:**
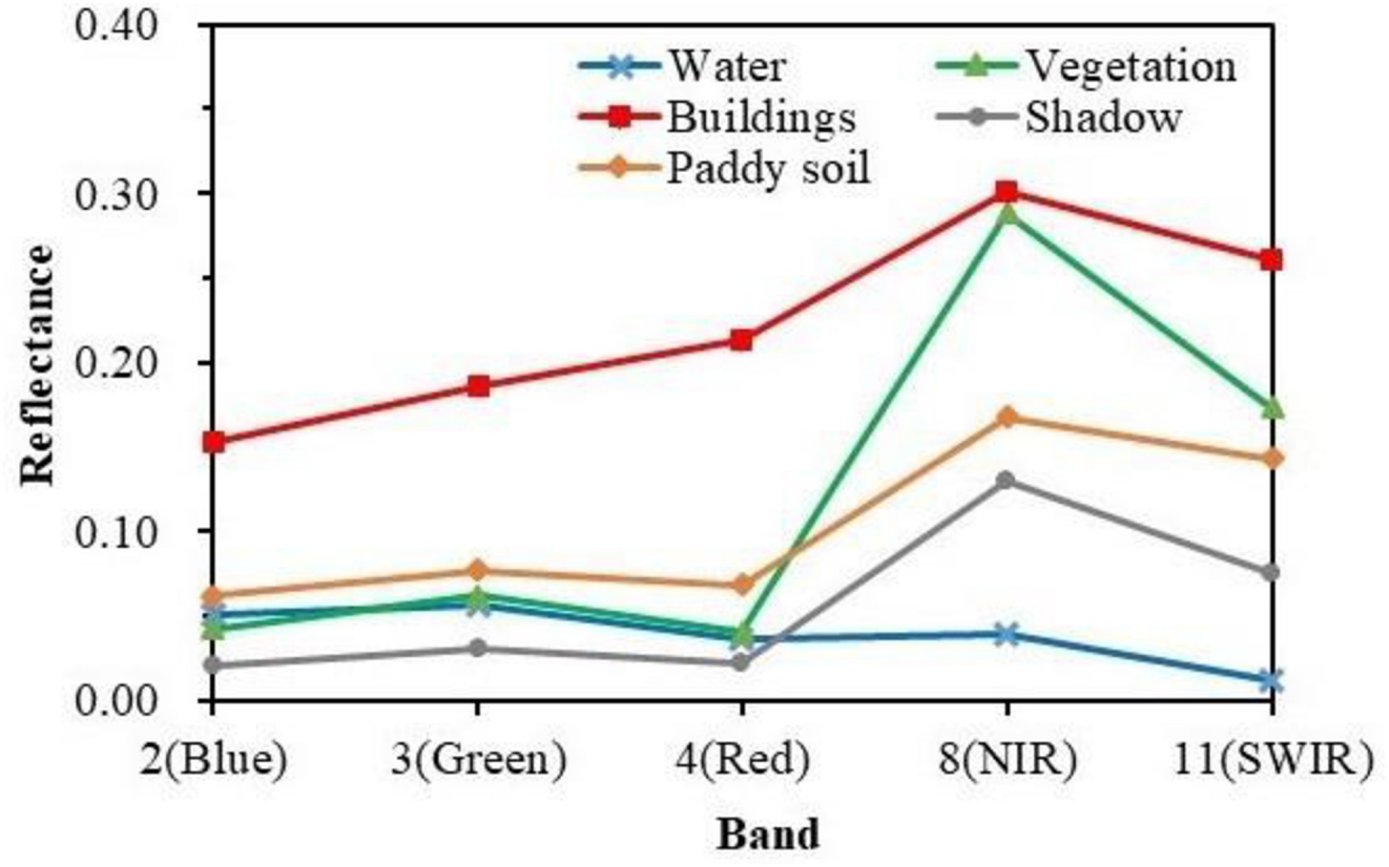
Spectral characteristic curve of typical features.

Aiming at water, shadow, paddy soil, vegetation, and building, 50 samples were selected respectively in the study area 1, and the average reflectance of each typical feature in 2 (Blue), 3 (Green), 4 (Red), 8 (NIR) and 11 (SWIR) bands of Sentinel-2 image was calculated. Reflectance refers to the ratio of the luminous flux reflected by an object to the incident luminous flux in a certain band. The greater the difference of reflectance between different objects in the same spectral band, the easier it is to distinguish. The spectral reflectance characteristic curve can reveal the difference of different objects in the same band. Fig 6 shows the spectral characteristic curves of five typical features. In the visible light band, the reflectance of water is high in the blue and green band, and low in the red band. In the near-infrared and short-wave infrared band, the reflectance of the water is low, and almost all incident energy is absorbed. The reflectance of the water shows a decreasing trend, namely: Blue > Green > Red > NIR > Short-wave infrared. The spectral characteristic of the shadow is very similar to that of the water. Their reflectance intersects in the red band, and is close to each other in other bands, and the discrimination is low. Mountain shadow and cloud shadow mainly exist in the study area, which affect the accuracy of water extraction. Because of the high water content and low reflectance of paddy soil, it is dark in remote sensing image, which will affect the extraction results of water. The spectral curves of building and vegetation are quite different from those of water, so they are easy to distinguish.

### Comparison of results of different water extraction models

**Fig 7:**
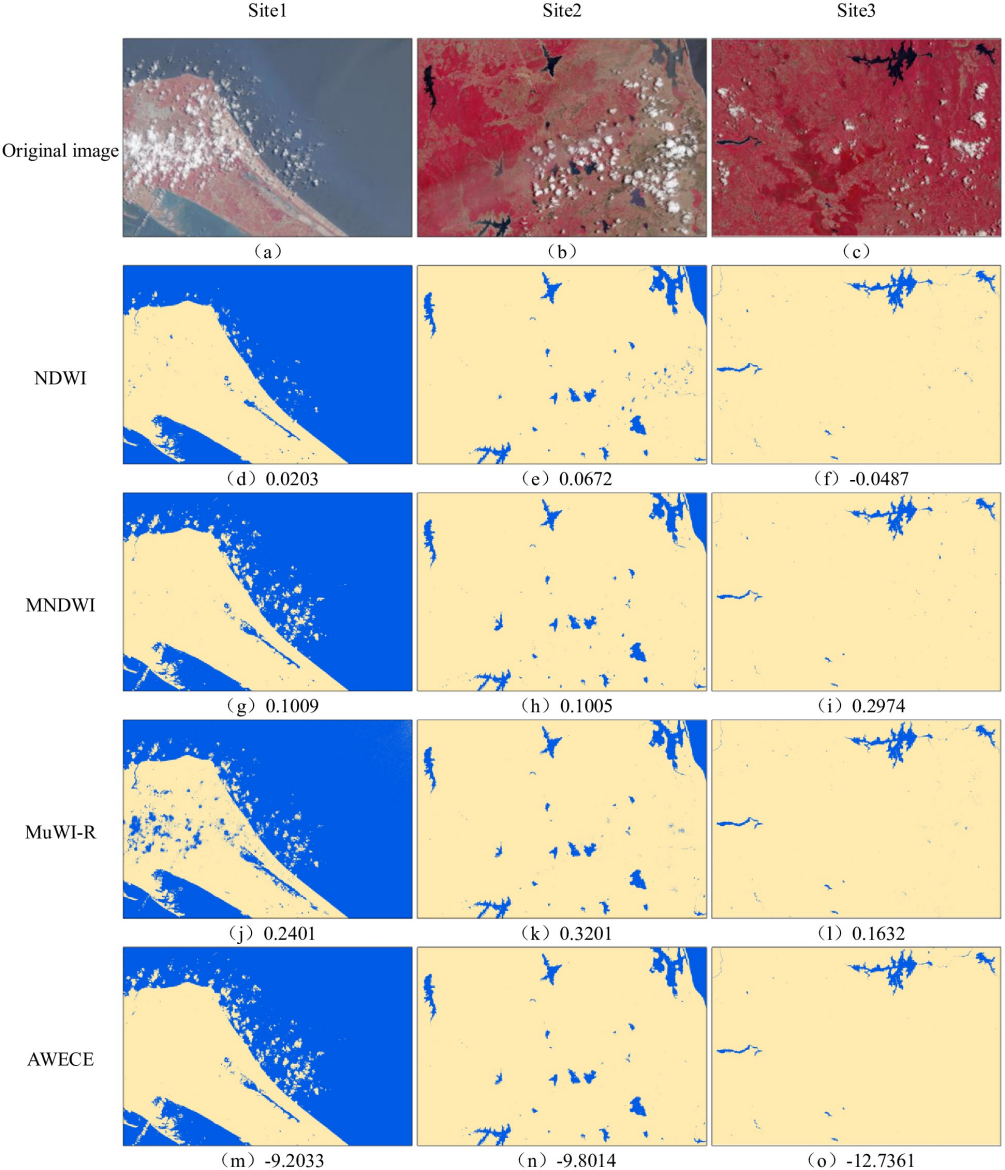
Water extraction results of different models. (a)-(c) are the original images of the three sites. Water extraction results of, (d)-(f) NDWI, (g)-(i) MNDWI, (j)-(l) MuWI-R and, (m)-(o) are the results of water extraction of NDWI, MNDWI, MuWI-R and AWECE respectively. The number after (d)-(o) is represents the threshold determined by the OTSU algorithm.

Fig 7 shows the water extraction results of different models in the most typical areas of the three sites. By comparing the water extraction results with the original images, it is found that the water extraction performance of NDWI is poor, and there are a lot of cloud shadows in the results of the three sites. At the same time, the results of site 2 include some paddy soils, and the southern area of site 3 contains some mountain shadows with discrete distribution. Compared with NDWI, the water extraction performance of MNDWI has been greatly improved, and the results of site 2 contain only a small amount of cloud shadow and paddy soil. However, the performance of MNDWI is not as good as that of NDWI in the extraction test of small rivers in the northwest of station 3. There are a large number of omissions in the results, and the river is extremely discontinuous. In the water extraction performance test of three sites, MuWI-R is extremely unstable. The results of site 1 contain a large number of clouds and cloud shadows, while the site 2 contain a small amount of cloud shadows and paddy soils. The results of site3 are better, only a very small amount of cloud shadows are included, and the extraction results of small rivers are similar to those of NDWI. The result of site 3 is good, which contains only a few cloud shadows, and the result of small river is similar to that of NDWI. AWECE performed well in the water extraction results of the three sites, which can avoid the effects of cloud shadow, mountain shadow and paddy soil, and the details of water extraction results are better.

To further compare and analyze the water extraction performance of different models, 500 samples were randomly selected based on the visual interpretation results of each image. Through the calculation of confusion matrix [39], the extraction accuracy of water is reflected from three aspects of omission error, commission error and overall accuracy, and the statistical results are shown in Table 4. It can be seen from the table that the overall accuracy of NDWI is low, and there are high omission error and commission error. The overall accuracy of MuWI-R is low because of the large number of mis-extraction results in site 1, but the omission error is low at only 2.18%. The overall performance of MNDWI is average, with an overall accuracy of 88.29%. Compared with the other three spectral water index methods, the overall accuracy of AWECW has been greatly improved, reaching 97.16%, and the omission error and commission error is very low.

**Table 4:** The accuracy of different water extraction models.

Model	Omission error	Commission error	Overall accurancy
NDWI	7.21	18.22	80.36
MNDWI	4.17	10.76	88.29
MuWI-R	2.18	19.89	81.45
AWECE	0.74	2.35	97.16

## Discussion

**Fig 8:**
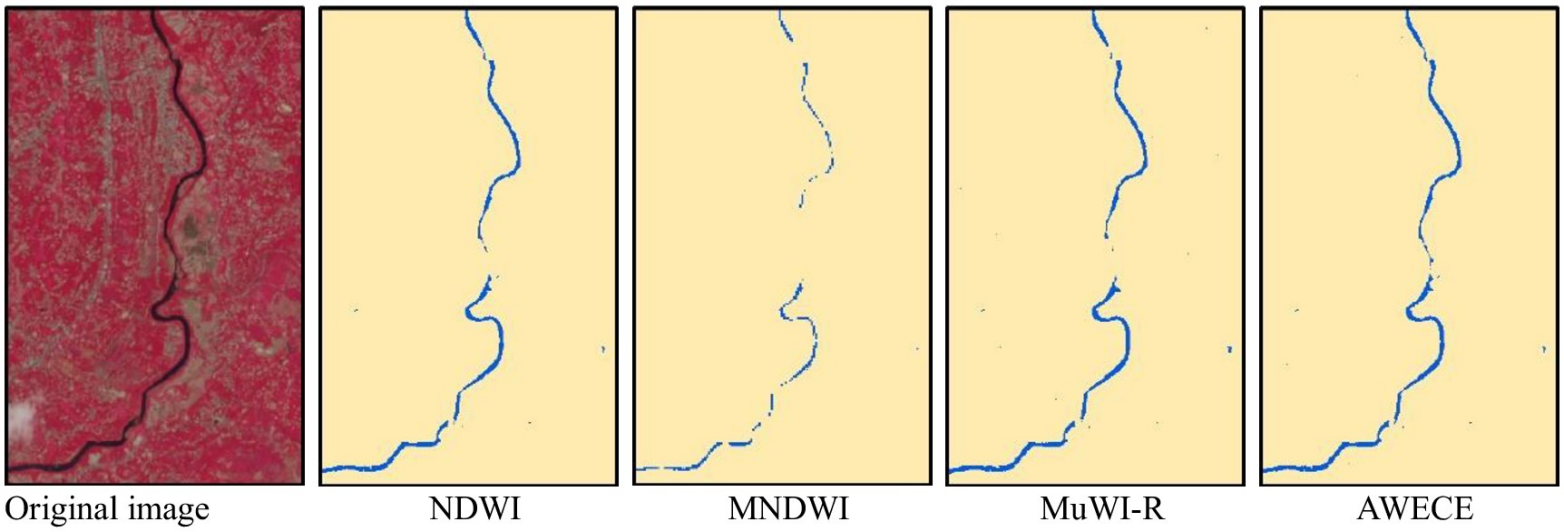
Extraction results of small river by different models.

The reflectance of water in short-wave infrared band is very low, and almost all the incident energy is absorbed, so it is often used as the input band of spectral water index method [8, 9, 40]. However, the resolution of the two short-wave infrared bands of Sentinel-2 image is 20m, which affects the fineness of water extraction results to a certain extent [19, 20]. Fig 8 shows the extraction results of small river in the northwest of site 3 by different models. As can be seen from the picture, the river extracted by MNDWI is extremely discontinuous, because the input band contains a short-wave infrared band with a resolution of 20m, while the minimum width of the river is only 1 or 2 pixels. It has been proved that the performance of water extraction of NDWI is weaker than that of MNDWI [17, 41], but the input band of NDWI in Sentinel-2 image is 10m, so the detail of the extracted river is stronger than that of MNDWI. MuWI-R selects four 10m bands and two 20m bands as input features, and trains through SVM to find the optimal separation hyperplane to determine the water index parameters. At the same time, the weight of the 10m band is relatively large, so it can make full use of the information of 10m band to extract water. Compared with MNDWI, the small river extracted by MuWI-R is more complete. However, the machine learning algorithm is lazy to the samples, and the training samples of MuWI-R are limited, so its stability is insufficient. AWECE has the best performance in the small river extraction test, because more bands participate in the calculation to enhance the characteristics of the water, so it ensures the continuity and integrity of the river.

**Fig 9:**
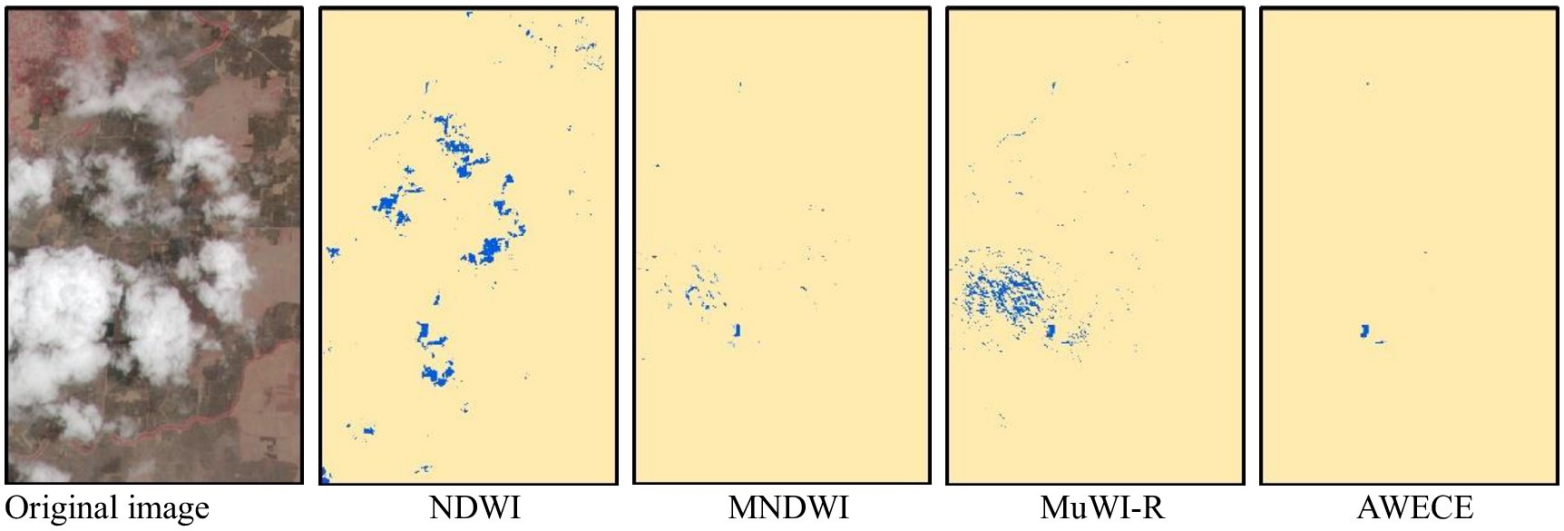
Water extraction results of different models under extremely complex conditions.

Fig 9 shows the water extraction results of different models in the extremely complex area of site 2. As can be seen from the original image, the area is covered by a large number of clouds and paddy soils. The results of NDWI include a large number of cloud shadows and a small amount of paddy soils. The results of MNDWI contains a small number of clouds and cloud shadows, as well as tiny amounts of paddy soils. The results of MuWI-R include a large number of clouds and tiny amounts of cloud shadows. Since AWECE includes cloud mask processing of the image, its water extraction performance is only affected by cloud shadows and paddy soils. There are great differences in the wetness component of K-T transformation among water, paddy soil and shadow, which ensures the water extraction performance of AWECE under extremely complex conditions.

## Conclusions

A fully automated water extraction model in complex environment (AWECE) based on Google Earth Engine and Sentinel-2 images was built through this study. To test the water extraction performance of different models, three sites in Sri Lanka were chosen. The accuracy of AWECE and three other types of spectral water index methods (NDWI, MNDWI, and MuWI-R) were evaluated from the perspective of visual interpretation and quantitative analysis. The results show that NDWI has serious commission errors, MNDWI has poor results in extracting small rivers, and MuWI-R lacks stability. Compared with the other three spectral water indices, the overall accuracy of AWECE is the highest, reaching 97.16%, which effectively avoids the effects of cloud shadows, mountain shadows, and paddy soils on water extraction. The AWECE model provides an effective solution for the precise extraction of surface water in complex environments and has important practical significance for water resource investigation, monitoring, and protection.

## Supporting information

S1 Dataset Sentinel-2 image metadata information and download link. (7Z)

S2 Dataset Shapfiles of image coverage of three sites. (7Z)

S3 Dataset OTSU, cloud mask and K-T transformation Google Earth Engine code. (7Z)
